# Loss of ORP3 induces aneuploidy and promotes bladder cancer cell invasion through deregulated microtubule and actin dynamics

**DOI:** 10.1007/s00018-023-04959-6

**Published:** 2023-09-22

**Authors:** Xue Wang, Junnan Liu, Anca Azoitei, Tim Eiseler, Sabine Meessen, Wencheng Jiang, Xi Zheng, Arika W. Makori, Markus Eckstein, Arndt Hartmann, Stephan Stilgenbauer, Mohamed Elati, Meike Hohwieler, Alexander Kleger, Axel John, Friedemann Zengerling, Felix Wezel, Christian Bolenz, Cagatay Günes

**Affiliations:** 1 Department of Urology, Ulm University Hospital, Helmholtzstr. 10, 89081 Ulm, Germany; 2 Department of Internal Medicine I, University Hospital, Ulm, Germany; 3 Institute of Pathology, Friedrich-Alexander University, Erlangen, Germany; 4 Department of Internal Medicine III, University Hospital, Ulm, Germany; 5 CANTHER, ONCOLille Institute, University of Lille, CNRS UMR 1277, Inserm U9020, 59045 Lille Cedex, France; 6 Institute of Mol. Oncology and Stem Cell Biology, University Hospital, Ulm, Germany; 7 Present Address: Department of Urology, Mayo Clinic College of Medicine and Science, Rochester, MN USA; 8 Present Address: Molecular Pharmacology and Experimental Therapeutics, Mayo Clinic College of Medicine and Science, Rochester, MN USA; 9 Present Address: Division of Hepatology, Department of Medicine II, Medical Faculty Mannheim, University of Heidelberg, Heidelberg, Germany; 10 Department of Urology, Nanjing Drum Tower Hospital, Nanjing University Medical School, Nanjing, 210008 Jiangsu China; 11 Institute of Urology, Nanjing University, Nanjing, 210008 Jiangsu China

**Keywords:** OSBPL3, Spindle orientation, Chromosomal imbalance, Invadopodia, Metastasis, Urothelial carcinoma

## Abstract

We have recently shown that loss of ORP3 leads to aneuploidy induction and promotes tumor formation. However, the specific mechanisms by which ORP3 contributes to ploidy-control and cancer initiation and progression is still unknown. Here, we report that ORP3 is highly expressed in ureter and bladder epithelium while its expression is downregulated in invasive bladder cancer cell lines and during tumor progression, both in human and in mouse bladder cancer. Moreover, we observed an increase in the incidence of N-butyl-N-(4-hydroxybutyl)-nitrosamine (BBN)-induced invasive bladder carcinoma in the tissue-specific Orp3 knockout mice. Experimental data demonstrate that ORP3 protein interacts with γ-tubulin at the centrosomes and with components of actin cytoskeleton. Altering the expression of ORP3 induces aneuploidy and genomic instability in telomerase-immortalized urothelial cells with a stable karyotype and influences the migration and invasive capacity of bladder cancer cell lines. These findings demonstrate a crucial role of ORP3 in ploidy-control and indicate that ORP3 is a bona fide tumor suppressor protein. Of note, the presented data indicate that ORP3 affects both cell invasion and migration as well as genome stability through interactions with cytoskeletal components, providing a molecular link between aneuploidy and cell invasion and migration, two crucial characteristics of metastatic cells.

**Supplementary Information:**

The online version contains supplementary material available at 10.1007/s00018-023-04959-6.

## Introduction

ORP3 (Oxysterol-Binding Protein-related 3; also known as OSBPL3 for OSBP-like 3), encoded by the *OSBPL3* gene, is a member of the oxysterol-binding protein (OSBP) family of proteins (OSBP and ORP1-11) [1, 2]. The OSBP-related proteins (ORPs) have been shown to mediate oxysterol metabolism and cellular signal transduction as well as vesicle transport [3]. All ORPs share a C-terminal oxysterol binding domain (i.e. oxysterol-related domain:ORD), which binds cholesterol derivatives and other ligands, such as phosphatidylinositol phosphates (PIPs) [4, 5]. Based on their structure, ORPs are classified in six different subgroupes, while ORP3 together with close homologues ORP6 and ORP7 belongs to the ORP subgroup III. This class of ORPs share a pleckstrin-homology (PH) domain (PHD) and an FFAT motif (two phenylalanine in an acidic tract) as functional domains [6]. ORP3 is targeted to the plasma membrane by its PH-domain, while its FFAT motif targets it to the endoplasmic reticulum (ER) [7–9]. Recruitment of ORP3 to the plasma membrane is stimulated by protein kinase C (PKC) activation and is determined by the plasma membrane phosphoinositides PI(4,5)P2 and PI4P [10].

Aside from their role in lipid and vesicle transport, several ORPs have been linked to cancer [11–15]. However, relatively little is known about the molecular mechanisms by which ORPs contribute to carcinogenesis. Previous reports indicated that ORP3 is associated with cell adhesion, invasion and migration [8, 9]. Notably, we previously identified a role of ORP3 in ploidy-control and provided evidence for a tumor suppressor function of this ORP [16, 17]. The knockdown of ORP3 induced aneuploidy in several cell types of different tissue origin and promoted the tumorigenic conversion of telomerase positive primary human fibroblasts in combination with SV40-LT-Ag. In addition, ORP3 knock out led to aneuploidy induction and tumor formation in mice [17]. However, the precise molecular mechanisms by which ORP3 impacts ploidy-control, cancer initiation and progression have yet to be revealed.

In the present study, we determined *ORP3* expression in a broad range of human tissues and found that *ORP3* is highly expressed in bladder and ureter epithelium while its expression is downregulated in invasive bladder cancer cell lines and during tumor progression, both in human and in mouse BC. ORP3 knockdown resulted in aneuploidy induction and genome instability in human ureter-derived epithelial cells with normal karyotype and increased migration and invasion capacities of the NMIBC cell line RT4. Conversely, ectopic ORP3 expression impaired migration and invasion capacities of BC cell lines. Pull-down and immunocytochemistry experiments revealed that ORP3 interacts with γ-tubulin and F-actin. Fluorescence recovery after photobleaching (FRAP) and live cell imaging experiments demonstrate that loss of ORP3 modulates the dynamics of actin and microtubules, respectively. In summary, the presented data indicate that loss of ORP3 affects proper chromosome segregation through impairing proper γ-tubulin function, leading to loss of ploidy control. On the other hand, the impairment of ORP3 actin cytoskeleton interactions in urothelial cells and bladder cancer cell lines have consequences on their migration and invasion capacities. These findings are relevant in the field of cancer because they demonstrate a molecular link between elevated aneuploidy, tumor cell invasion and metastatic cascade.

## Materials and methods

### Human cell lines

HEK293T cells were cultured in DMEM (Gibco-ThermoFisher, MA, USA), supplemented with 10% fetal calf serum (FCS) (Sigma-Aldrich, St. Louis, USA) and 1% penicillin/streptomycin (P/S) (PAN Biotech, Aidenbach, Germany). The simian virus 40 (SV40) large T antigen immortalized UROtsa cells were a gift from Dr. Phillip Erben, Mannheim, Germany. Y235T cells were a gift from Prof. Jennifer Southgate (University College London, UK). HBLAK cells were a gift from Michele Hoffmann (Department of Urology, Düsseldorf, Germany). Y235T and HBLAK cells were cultured in a complete epithelium culture medium, CnT-Prime (CELLnTEC, Bern, Switzerland). UROtsa cells were cultured in RPMI1640 (Gibco-ThermoFisher, MA, USA) supplemented with 1% GlutaMAX (Gibco-ThermoFisher, MA, USA) and 1% non-essential amino acids (Merck Millipore, Darmstadt, Germany), 10% FCS and 1% P/S. UMUC3, RT4 and T24 were cultured in RPMI1640, supplemented with 10% FCS and 1% P/S. All cell lines were kept at 37 °C in a humid atmosphere with 5% carbon dioxide.

### Transfection, infection and generation of stable cell lines

DNA transfection was carried out by using polyethylenimine (PEI) (Cat. 23966, Ploysciences, Warrington, USA) according to supplier’s recommendations. For virus production, HEK293T cells were transfected at 40–50% confluency and incubated for 24 h prior to transfection (for lentiviral vectors: 30 µl PEI, 1.8 µg psPAX2, 0.3 µg pMD2.G and 3 µg target plasmid were mixed with 500 µl of DMEM medium without FCS on the day of transfection; for retroviral vectors, 30 µl PEI, 1.8 µg pCMV-Gag-Pol, 0.3 µg pCMV-VSV-G and 3 µg target plasmid were mixed with 500 µl of DMEM medium without FCS). Virus collection and cell infection was performed according to standard procedures. For generation of cells with stable target gene expression, cells were permanently kept under selection with 100.0 µg/ml Zeocin (ThermoFisher Scientific, Waltham, USA), 2.0 µg/ml Puromycin (ThermoFisher Scientific, Waltham, USA), 25.0 µg/ml Hygromycin (ThermoFisher Scientific, Waltham, USA) or 12.5 μg/ml Blasticidine (ThermoFisher Scientific, Waltham, USA), respectively.

### Expression plasmids

The pcDNA4/HisMaxC-ORP3 and pEGFP-ORP3 vectors were gifts from Prof. Vesa M. Olkkonen (National Public Health Institute, Finland). LifeAct-Ruby was a gift from Dr. Tim Eiseler (Ulm University Hospital, Ulm, Germany). EB3-GFP was a gift from Prof. Holger Bastians (University of Göttingen, Germany). The EB3-GFP ORF was subcloned into the EcoRI/SalI digested pBABE-puro vector. Cloning primers for the subcloning are shown in Supplementary Table 1.

### Total RNA, reverse transcription and quantitative real-time PCR (RT-qPCR)

Human Total RNA Master Panel II was purchased from Clontech (Cat. No. 636643, Clontech Laboratories, California, USA). RNA from cultured cells were isolated as follows: 1 × 10^5^ cells were seeded into T25 flasks. After two days growth, cells were washed with 1xPBS and added with 350 µl RLT buffer (Qiagen, Hilden, Germany) containing 1% β-mercaptoethanol (Sigma-Aldrich, St. Louis, USA). Total RNA was isolated by the RNeasy^®^ mini kit (Qiagen, Hilden, Germany) according to the manufacturer’s protocol. The concentration and purity were measured by the Epoch microplate spectral photometer (Biotek Instruments, Winooski, USA) at 260 nm and 280 nm wavelength, and 100 ng total RNA was used for reverse transcription with the GoScriptTM reverse transcription kit (Qiagen, Hilden, Germany). For quantitative PCR, 2 µl of 1:10 diluted cDNA used. Applied biosystem Viia7 Real Time PCR system (Applied biosystems, Foster City, USA) and MicroAmp fast optical 96-well reaction plate was used for RT-qPCR experiment. The quantitative PCR was run in Viia7 cycler according to the conditions shown in Supplementary Table 4. The relative expression of target genes was evaluated by ΔCT. *GAPDH* was used as the reference gene for detection of relative mRNA levels. Primer sequences and the amplified products length are listed in Supplementary Table 4.

### Protein extraction and western blot

For protein expression analyzes, 5 × 10^5^ cells were seeded in 10 cm plates and harvested at 70–80% confluency by scraping with 150 µl cells lysis buffer (Cell Signaling Technology, Danvers, USA) containing phosphatase and protease inhibitors (Roche, Rotkreuz, Germany). Then the cell lysate solution was centrifuged at 14000 rpm for 25 min at 4 °C and the supernatant was collected. The protein concentration was determined by Bicinchoninic acid (BCA) assay (Sigma-Aldrich, St. Louis USA). 40–200 µg of protein samples were loaded on a 10% acrylamide gel and the immunoblot was performed according to standard procedures.

### Antibodies

Anti-ORP3 rabbit antibody (1:2000 for WB and 1:400 for IF, A304-557A, Bethyl Laboratories, Texas, USA). Anti-ORP3 mouse antibody (1:400 for IHC and 1:2000 for WB, sc398326, Santa Cruz, California, USA). Anti-Cortactin mouse antibody (1:500 for IF, sc-55579, Santa Cruz, California, USA). Anti-α-tubulin mouse antibody (1:2000 for WB, T5168, Sigma-Aldrich, St. Louis, USA). Anti-γ-tubulin mouse antibody (1:2000 for WB and 1:500 for IF, T5326, Sigma-Aldrich, St. Louis, USA). Anti-γ-tubulin rabbit antibody (1:500 for IF, T5192, Sigma-Aldrich, St. Louis, USA). Anti-β-actin mouse antibody (1:10,000 for WB, A1978, Sigma-Aldrich, St. Louis, USA). Secondary antibodies, HRP anti-Mouse antibody (1:5000 for WB, 7076S, Cell Signaling Technology, Danvers, USA) and HRP anti-Rabbit (1:2000 for WB, 7074S, Cell Signaling Technology, Danvers, USA). One drop of Universal immune-peroxidase anti-Mouse (Nichirei Bioscience, Tokyo, JP) per piece of tissue was used in IHC. Phalloidin-iFluor 488 Reagent (1:1000 for IF, ab176753, Abcam, Cambridge, UK). Phalloidin-iFluor647 reagent (1: 1000 for IF, ab176759, Abcam, Cambridge, UK). Anti-Rabbit conjugated with Alexa Fluor488 antibody (1:1000 for IF, A11008, ThermoFisher Scientific, Waltham, USA), and anti-Mouse conjugated with Cy5 antibody (1:200 for IF, 115–605-006, ThermoFisher Scientific, Waltham, USA).

### N-butyl-N-(4-hydroxybutyl)-nitrosamine (BBN)-induced carcinogenesis

BBN-treatment was essentially performed as described [15]. Briefly, mice received 0.1% BBN (Sigma Aldrich Biochemie GmbH, Hamburg, Germany. Cat. Nr. B8061*) *ad libitum in drinking water up to 5 months. The drinking water containing BBN was prepared once a week. The experiments were conducted with the approval of the Baden Württemberg animal ethics committee (animal experiment number: (35/9185.81-3/1326)). For histopathological visualization 2 μm thick whole slide section of each FFPE block were cut, mounted on conventional STARFROST glass slides and stained with Hematoxylin and Eosin (HE).

### Orp3 conditional knockout mice

*Orp3* (*Osbpl3*) mice (Osbpl3^tm1a(EUCOMM)Wtsi^) were provided by the International Mouse Phenotyping Consortium (IMPC). Mice with the *Orp3* targeted allele containing two intronic FRT sites flanking a LacZ gene with an internal ribosome entry site (IRES) and a neomycin resistance (Neo) cassette were crossed with mice expressing the Flp recombinase to generate the floxed-Orp3 (Orp3^fl/fl^) mice according to the IMPC suggestion. The floxed Orp3 mice were then crossed with K14-Cre^tg^ mice (B6. KRT14^(cre)1Amc/J^, a gift from Dr. Karin Scharffetter-Kochanek, Ulm) to generate *Orp3* knockout (*Orp3*^*−/−*^) mice under the control of the keratinocyte 14 gene promoter. The animals were housed under the following controlled conditions: a 12 h light/12 h dark cycle, a steady temperature of 25 ± 1 °C, and with free access to water and food. The animal experiment was approved by local research ethics committee (35/9185.81-3/1326). The experimental procedures were conducted according to the ethical guidelines for the care and use of laboratory animals of the National Institutes of Health (https://grants.nih.gov/grants/olaw/guide-for-the-care-and-use-of-laboratory-animals.pdf) and the International Association for the Study of Pain (IASP). Every effort was made to decrease the number of animals used and to reduce animal suffering. Genotyping was performed by PCR assay with genomic DNA extracted from tail biopsies after weaning using the Mouse Direct PCR Kit (B40015, Bimake, TX, USA). Genotyping primers are provided in Supplementary Table 5.

### Immunohistochemistry

Hematoxylin and eosin stainings, initial tests to establish IHC conditions, and the identification of the target proteins in human ureter and in the human and murine BC samples were carried out in our lab at the University of Ulm using 4 µm tissue sections in accordance with established protocols. IHC samples were stained with 3,3’-Diaminobenzidine (DAB) substrate solution (Scytek Laboratories, West Logan, USA). The pictures were acquired by using Zeiss TCS SP5 confocal microscope (Zeiss, Oberkochen, Germany) with 10 × and 63 × objectives. IHC experiments to evaluate ORP3 expression in 190 MIBC, as well as the in 26 normal human bladder samples were performed at the University Hospital Erlangen. An expert pathologist (Dr. M. Eckstein) stained the samples and assessed signal intensities using Axio Imager A2 (Zeiss, Oberkochen, Germany). The MIBC samples are from a previously described, well-characterized MIBC cohort [18, 19] (see also Figure S2). Ethical approval for this study was obtained by the ethical review board of the Friedrich-Alexander-University Erlangen-Nürnberg (Erlangen, Germany; approval number: no. 3755 and 329_16B). All patients agreed and gave informed consent, and all analyzes were carried out in accordance with the Declaration of Helsinki.

### Patients samples collection and handling at Ulm University

For the two urothelium samples, ureters were collected from patients, who underwent a nephrectomy at the Urology Department, at the University of Ulm. In brief, a 5 mm piece of ureters was cut from the kidney separated from patients and put in DMEM medium immediately after surgery. After removing fat and fibrous connective tissue, ureters were opened with a scissor in a 10 cm plate containing 10 ml of culture medium and a scalpel was used to scrape the inner epithelial layer of the ureters. The detached epithelial cells were transferred into a 15 ml falcon and centrifuged at 1200 rpm for 3 min. The cell pellet was collected for subsequent RNA extraction. The studies were conducted with the patients' written consent and with the local research ethics committee's approval (239/18).

### Karyotype analyzes (chromosomes spread assay)

The chromosomes spread assays to determine the aneuploidy induction were conducted after 5–7 days selection with puromycin following the infection with virus containing the ORP3-targeting shRNA vectors to eliminate the non-infected cells. The Y235T cells were then seeded on coverslips in 6-well plates. After 48 h, cells were treated with 10 µg/ml KaryoMAX^®^ Colcemid (ThermoFisher Scientific, Waltham, USA) for 4–6 h and washed three times with 1xPBS and incubated 20 min in 2 ml of 0.075 M KCl at 37 °C. Cells were then fixed with cold metaphase fixation solution (acetic acid: methanol 1: 3) for 15 min. After adding a drop of DAPI mounting medium (ThermoFisher Scientific, Waltham, USA) mounting medium on observation slides, the cover slides were reversed and placed on the drop of DAPI. The slides were dried under dark room overnight and analyzed under the Zeiss TCS SP5 confocal microscope with a 100 × objective.

### Detection of micronuclei and multinucleated cells

Y235T cells were cultured in a 12-well plate with a 19 mm Ø round coverslip for at least 48 h without any treatment. Cells were then fixed with 4% PFA and stained with DAPI to visualize cell nuclei and with Phalloidin-AF488 to visualize the cell membrane. For each slide, more than 1000 cells were counted. The pictures were captured by using the Zeiss TCS SP5 confocal microscope with a 63 × objective.

### Detection of lagging chromosomes

Y235T cells were cultured in a 12-well plate with a 19 mm Ø round coverslip for at least 48 h without any treatment. Cells were extracted with microtubule stabilization buffer (MSB) for 5 min, followed by 10 min fixation with 4% PFA. In alternative experiments, cells were fixed for 3 min with ice-cold methanol without MBS treatment. Cells were stained with α-tubulin specific antibody to visualize spindles and with γ-tubulin specific antibody to visualize spindle poles, and with DAPI to visualize chromosomes. A total of 270–710 cells in mitosis from 3–4 independent experiments were counted. The pictures were acquired with the Zeiss TCS SP5 confocal microscope using a 63 × objective.

### Determination of spindle orientation

This experiment was performed according to Stolz et al. [20]. Y235T cells were seeded in 12 well-plates with cover slips. After 4 h of treatment with 2 µM Dimerthylenastron (DME) (Merck Millipore, Darmstadt, Germany), the cells were fixed with ice-cold methanol for 6 min. Fixed cells were stained for α-tubulin, γ-tubulin and with DAPI to visualize metaphase spindles, centrosomes and chromosomes, respectively. Microscopy was performed with a Zeiss TCS SP5 confocal microscope equipped with a Zeiss Apotome 2 (Zeiss, Oberkochen, Germany) using a 63 × objective. Images were recorded with a Z-optical spacing of 0.2–0.4 μm. Spindle orientation was determined by measuring the angle between the centrosome axis and the growth surface following mathematic formula yielding the spindle pole displacement factor (SPDF), which is calculated by the distance from the cell center to the spindle pole divided by the radial distance from the center of the cell to the cell border [20].

### Microtubule plus-end tracking with EB3-eGFP

Microtubule plus-end tracking was performed as previously described [21]. To visualize microtubule polymerization at plus-ends, End-binding-protein EB3 tagged with enhanced GFP (EB3-eGFP) was stably expressed in Y235T cells. For live-cell imaging, cells were seeded onto 35 mm glass-bottom µ-dishes (Ibidi, Martinsried, Germany). Live-cell imaging was performed using a Zeiss LSM 710 microscopy with a 63 × objective as part of a Zeiss Cell Observer System with a large incubation chamber at 37 °C. In each group, video images were acquired at one frame per three seconds. 30 frames were captured in 20 cells. In each cell, 20 particles per condition were tracked for 4 frames by using the ImageJ manual tracking tool [22] over a time span of 12 s and velocities were quantified.

### In vitro microtubule-binding assay

For GFP-ORP3 purification, 30 cell culture dishes with HEK293T cells, which were transiently transfected with a GFP-ORP3 expression vector, were collected at a confluency of 70% and total cell lysates were prepared with EDTA-free lysis buffer (20 mM Tris, pH 7.4, 150 mM NaCl, 5 mM MgCl2), supplemented with 1% Triton X-100 plus complete protease inhibitors (ThermoFisher Scientific, Waltham, USA). Lysates were clarified by centrifugation at 14,000 × rpm for 10 min. GFP-ORP3 purification was performed with 500 μl of GFP-Trap GFP-Trap (ChromoTekm, Planegg, Germany) according to the manufacturer's protocol. After extensive washing steps with lysis buffer, bound GFP-ORP3 protein was eluted with 3 × 1 ml of 150 mM glycine (pH 2.3) and collected in a 15 ml tube with 1 ml 1.5 M Tris (pH 8.8) buffer to neutralize the acid. Subsequently, eluates were concentrated using 10,000 MWCO Vivaspin2 centrifugal filtration devices (Sartorius Stedim Biotech, Laupheim, Germany) and rebuffered in TBS. GFP-ORP3 concentrations were subsequently assessed by Bradford assay and purity was tested by SDS-PAGE. Microtubule binding studies were performed as previously described [21] using the microtubule binding protein spin down assay kit (Cytoskeleton #BK029) according to the manufacturer’s protocol. In brief, the purified GFP-ORP3 was diluted with 10 × General tubulin buffer supplemented with 1% Triton X-100 plus. After centrifugation at 100,000×g for 30 min at 4 °C, the supernatant was kept for further use to get rid of the lipid associated fraction. The supernatant and the pellet fractions were analyzed by Western blot.

### Immunofluorescence

Cells were seeded and grown in a 12-well plate with a 19 mm Ø round coverslip in each well, and cultured for at least 48 h. For α-tubulin and γ-tubulin related staining, cells were extracted with microtubules stabilization buffer (MSB) for 5 min and then followed by 10 min fixation with 4% PFA or directly fixed in 3 min cold methanol without extraction. For F-actin related staining, cells were fixed with 4% PFA for 10 min. Then followed by two times wash with 1xPBS, 1% Triton X-100 in 1xPBS was used to permeabilized cells. After three times wash with 1xPBS, the cells were blocked with 1% BSA in 1xPBS at room temperature for one hour. Coverslips with cells were then incubated with primary antibody solution diluted in 1xPBS with 1% BSA over night at 4 °C. Next day, followed by three times wash in PBST (1xPBS containing 0,5% Tween), slides were incubated with an appropriate secondary antibody. Phalloidin reagent was mixed with secondary antibody and incubated with cells to visualize F-actin. Finally, samples were mounted with DAPI fluorescence mounting medium. Images were acquired with Zeiss TCS SP5 confocal microscope using a 63 × objective for colocalization and invadopodia detection. For the calculation of colocalization, Image J 1.53t (National Institute of Health, Bethesda, MD, USA) was used to measure Pearson’s coefficient value.

### Wound healing and Boyden chamber assays

Evaluation of cell migration by Wound healing assay and cell migration/invasion by Boyden chamber assay were performed as described previously [23]. Pictures were acquired using a 5× objective in case of the Wound healing assay and 10× objective in case of the Boyden chamber assay, using a Zeiss TCS SP5 confocal microscope.

### Ex vivo porcine bladder invasion model

The experiments were essentially performed as described by Wezel et al. [24]. Pictures were acquired from 6 random areas of bladder tissues with 100× and 200× magnification using a Zeiss TCS SP5 confocal microscope in order to quantify the invasive capacity of cells. Then, the distance of cells to the closest surface was measured for the 50 deepest invasive RT4 cells or 100 deepest invasive UMUC3 cells by ZEN software and the mean of the distance was calculated.

### Gelatin degradation assay

This assay was performed as described by Diaz [25]. Here, 3 × 10^4^ RT4 or 1 × 10^4^ T24 cells were seeded in 12-well plates with coverslips, coated with Gelatin-FITC. The cells were incubated in complete RPMI1640 medium at 37 °C in a humidified atmosphere with 5% CO2 for 3 days. The cells were then fixed with 4% PFA for 10 min, permeabilized with 1×PBS containing 0.1% Triton X-100 and blocked with 0.3% BSA for 30 min. After washing with 1×PBS, F-actin was visualized by staining with Alexa Flour-647 Phalloidin for 1 h at room temperature. Nuclei were stained with mounting medium containing DAPI. Confocal microscopy analyzes were performed using Zeiss TCS SP5 confocal microscope with a 40× objective. Degradation area was measured by using the ImageJ program, and the normalized area was calculated by the area dividing the cells in the whole picture.

### FRAP imaging of LifeAct-Ruby fluorescence intensity

FRAP experiments were essentially performed according to the protocol described by Weeber et al. [26]. Here, Ruby-LifeAct was expressed in UMUC3 cells and Ruby-LifeAct-positive cells were enriched (due to the lack of a selection marker) by FACS sorting in the Core Facility of Cytometry (Ulm University, Ulm, Germany). The cells were seeded in a T25 flask following initial sorting. The cells underwent another round of sorting and enrichment after achieving 70% confluence. The percentage of positive cells reached more than 90% after 4 rounds of repeated enrichment procedures, and the cells were used in the subsequent experiments. UMUC3 cells were seeded in a 35 mm glass-bottom µ-dishes. FRAP experiments were performed using a Leica TSC-SP8-HCS confocal laser scanning microscope (Leica, Wetzlar, Germany) with 63 × objective using a tabletop incubator (Oko Labs, Ottaviano, NA, Italy) at 37 °C and 5% CO2. 512 × 512 pixels images with 200 Hz scan speed were acquired with a scan speed of 2.58 s per frame. After placing a bleach-ROI on peripheral actin structures, five pre-bleach frames were acquired before bleaching of lamellipodia structures with LifeAct-Ruby fluorescence using a 561 nm laser line (AOTF 100%). Subsequently, LifeAct-Ruby fluorescence recovery was monitored by measuring intensities within the bleach-ROI for 75 frames. FRAP was quantified by placing 3 equally sized circular sub-ROIs with the bleached area and calculating the average mean of ROI fluorescence intensities per cell.

### F-actin co-sedimentation assay

GFP-ORP3 was purified as described above. F-actin co-sedimentation assays were performed as previously described [27]. The purified GFP-ORP3 extracts were diluted 1:10 in F-buffer (10 mM imidazole, 75 mM MgCl2, 0.5 mM DTT, 1 mM EGTA, pH7.2) supplemented with 1% Triton X-100 plus. After centrifugation at 100,000×g for 20 min at 4 °C to remove the lipid associated protein fraction, the supernatant was collected. Rabbit muscle G-actin (AKL99, Cytoskeleton) was diluted in 500 µl F-buffer and then polymerized for 1 h at RT (2.5 mg/ml). 40 µl F-actin were incubated with 25 µl of purified GFP-ORP3 protein for 30 min at RT, followed by centrifugation at 100,000 × g for 1 h at RT. After the supernatant was transferred into 1.5 ml tubes, the pellet was incubated for 1 h in 200 µl G-buffer (5 mM Tris–HCl, pH 8, 0.5 mM DTT, 0.2 mM CaCl2, and fresh 0.2 mM ATP) to depolymerize F-actin. Equal amounts of supernatant and pellet fraction were analyzed by Western blot.

### Statistical analyzes

Statistical analysis and graphic representations were performed by using GraphPad Prism 6 or Excel. For average, the comparison between two groups were analyzed by using student’s t test. Data were tested to be normally distributed except for human IHC results shown in Fig. 4E, due to the low number of samples in the normal bladder (NB) group. Here, the significance was determined by Welch’s test with 95% confidence interval. The Log Rank Test was used to determine the overall survival or recurrence free survival of patients with BC. For standard deviation, F test was used. All values are expressed as median and standard error of the mean (SEM) unless otherwise stated. A value of p < 0.05 was considered statistically significant.

## Results

### ORP3 expression in human tissues

**Fig. 1:**
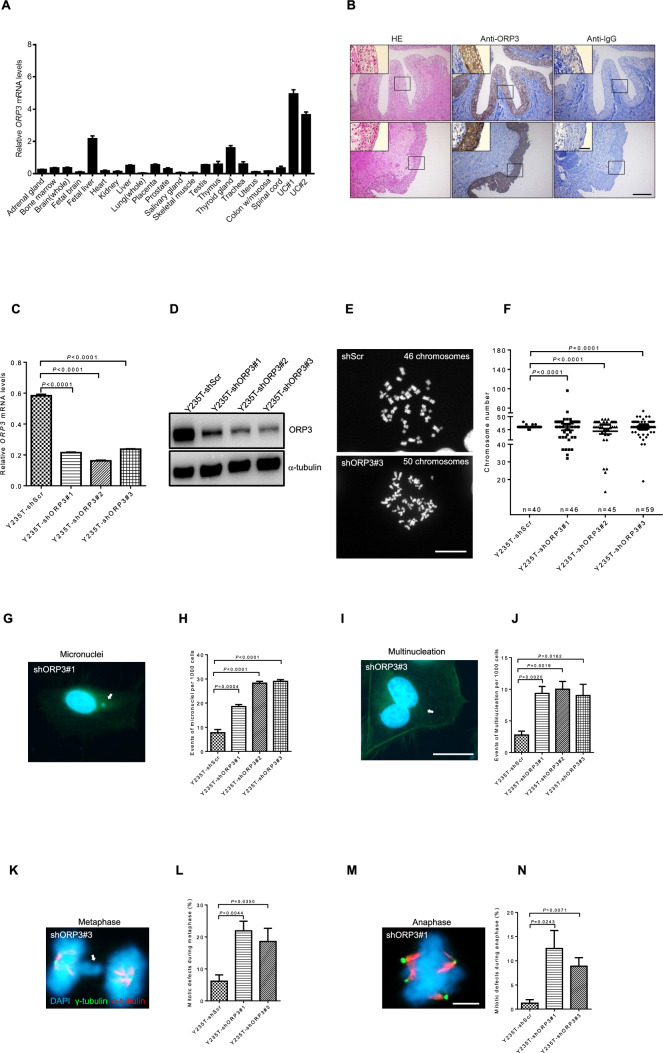
ORP3 control ploidy in Y235T cells. **A** The bar graph shows *ORP3* mRNA levels in the indicated human tissues (see Materials and Methods for detailed information). Total RNA of urothelial cells (UC#1 and UC#2, respectively) was prepared from ureter tissues of two distinct patients who have undergone nephrectomy at Ulm University. The mRNA level of each gene is calculated in reference to *GAPDH*. *n* = 3 independent experiments were performed. Error bars represent mean ± SEM.** B** The representative pictures indicate the OPR3 staining in human ureter tissues (UC#1 and UC#2, respectively). **C–D** Representative Western blot experiments displaying ORP3 protein levels in Y235T cells with shORP3. α-tubulin is used as a loading control. *n* = 3 independent experiments were performed. The bar graphs illustrate the efficiency of ORP3 knockdowns at the mRNA level in Y235T cells in reference to *GAPDH* mRNA level. The results are from *n* = 3 independent experiments. Error bars represent mean ± SEM.** E** Representative pictures displaying metaphase spreads of Y235T cells with shORP3#3. Chromosomes are visualized by DAPI. Control Y235T-shScr cells with 46 chromosomes in the majority of metaphase spreads. Exemplary pictures demonstrating the induction of aneuploidy in Y235T cells after ORP3 knockdown. The images show a metaphase spread of Y235T-shORP3#3 cells with 50 chromosomes. **F** Chromosomes numbers of metaphase spreads from Y235T cells that were infected with the indicated shRNAs targeting ORP3. *n* = numbers of each counting is indicated within the graph. Results are pooled from three independent sets of experiments. Mean ± SEM values are shown in the dot plot, and significance was determined by using Fisher’s exact test. **G-J** Representative pictures as well as quantification of micronuclei and multinucleation of Y235T cells with the indicated shRNAs targeting ORP3. Cells are stained nuclei with DAPI, and F-actin with phalloidin Alexa Fluor 488. Micronuclei and multinucleation are indicated with white arrows. Quantitative results from *n* = 4 independent sets of experiments. Mean ± SEM values are shown in the bar graph, and the significance was determined by two-tailed Student’s t test. **K–N** Mitotic defects in Y235T shORP3#1 and #3 cells during metaphase and anaphase. Cells were infected with the indicated shRNAs targeting ORP3. The γ-tubulin is illustrated by Alexa Fluor 488 (green) staining, α-tubulin is shown by Cy5 (red), and chromosomes are indicated by DAPI (blue). The misplacement of chromatids is marked with white arrows. Quantitation of mitotic defects Y235T cells with ORP3 knockdown during metaphase and anaphase, respectively. Results from *n* = 4 independent sets of experiments. Mean ± SEM values are shown in the bar graph, and significance was determined by two-tailed Student’s t-test. Scale bars: 200 μm (**B** main panels) 50 μm (**B** insets), 20 μm (**E**, **G**, **I**) and 5 μm (**K**, **M**). Images were captured at total magnification of 100 (**B** main panels) and 630 (**B** insets, **E**, **G**, **I**, **K**, **M**)

As a first attempt to gain further insight into the potential roles of *ORP3* in humans, its mRNA levels were determined by RT-qPCR in a set of commercially available normal human tissue samples and in epithelial cells derived from human normal ureter tissue. We find highest *ORP3* (Fig. 1A) mRNA levels in ureter-derived epithelial cells: low *ORP3* mRNA levels were detected in adrenal gland, heart kidney, lung, skeletal muscle, uterus, and spinal cord and moderate level of ORP3 found in placenta, prostate, testis, thymus, thyroid gland, and trachea. Strong ORP3 expression was detected at the protein level in human ureter and bladder epithelium (Fig. 1B and Supplementary Fig. 1A) as well as in murine bladder tissue (Supplementary Fig. 1B). These findings suggest that ORP3 may have an important function in the epithelial cells of the human and mouse ureter and bladder.

### ORP3 knockdown induces aneuploidy in Y235T cells

In order to explore the impact of ORP3 on genome stability in the urothelium, aneuploidy induction was determined by counting metaphase chromosome numbers. For this purpose, ORP3 levels were downregulated by three different shRNAs in Y235T cells, a telomerase-immortalized cell strain with a normal karyotype, derived from normal human ureter epithelium (Fig. 1C, D). Evaluation of metaphase spreads revealed an increased aneuploidy as a consequence of ORP3 knockdown in Y235T cells (Fig. 1E, F). Chromosome numbers in Y235T cells with ORP3 knockdown ranged from 32 to 96 in shORP3#1, 13 to 49 in shORP3#2, and 19 to 54 in shORP3#3. In contrast, the vast majority of control cells carrying a control shRNA vector (Y235T-shScr) had a normal karyotype. We also considered micronuclei formation and the presence of multiple nuclei in a single cell, as their appearance are also considered as indicators of aneuploidy and represent chromosomal instability. Micronuclei formation and multinucleation occurred more frequently in Y235T cells with ORP3 knockdown (Fig. 1G–J). The knockdown of ORP3 resulted by more than two–threefold increase of cells with micronuclei (Fig. 1G, H) and multinucleated cells (Fig. 1I, J). These results confirm earlier research from our group that indicated a role of ORP3 in ploidy-control and genome stability [16, 17].

Cells in various mitotic stages were examined more closely in order to comprehend the molecular mechanisms by which the downregulation of ORP3 results in the induction of aneuploidy. We observed an increased number of mitotic defects in response to ORP3 downregulation (Fig. 1K–N). ORP3 knockdown in Y235T cells resulted in approximately threefold increase of mitotic defects during metaphase (6.2% in shScr versus 21.9% in shORP3#1, *P* = 0.0044, and 18.6% in shORP3#3, *P* = 0.0350) (Fig. 1K, L), and seven to ninefold increase of mitotic defects during anaphase (1.2% in shScr vs 12.5% in shORP3#1, *P* = 0.0243, and 8.9% in shORP3#3, *P* = 0.0071) (Fig. 1M, N). These defects usually result from microtubule and/or kinetochore defects and indicate chromosome misalignments.

### ORP3 regulates spindle orientation and microtubule dynamics

**Fig. 2:**
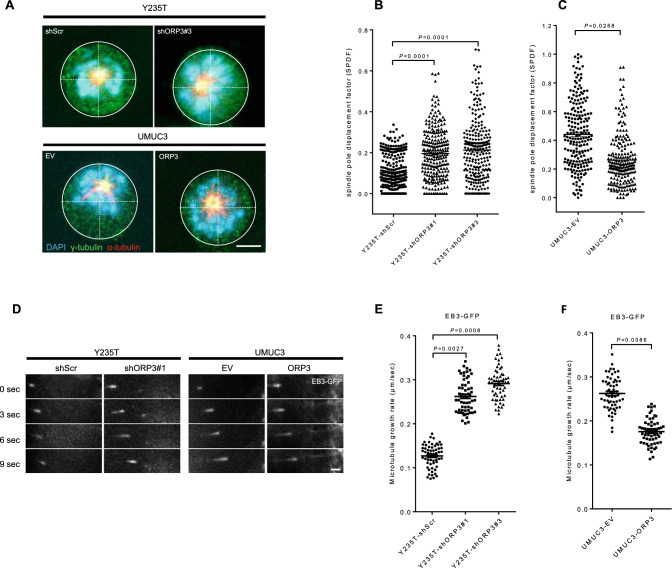
ORP3 control spindle orientation and microtubule dynamics. **A** Representative images displaying the disorientation of Y235T cells with shORP3#3 or reorientation of UMUC3 cells with ectopic ORP3. The γ-tubulin is illustrated by Alexa Fluor 488 (green) conjugated secondary antibodies, α-tubulin by Cy5 (red) staining, and chromosomes are indicated by DAPI (blue). **B–C** Quantitative evaluation of the spindle pole displacement factor (SPDF) upon knockdown of ORP3 in Y235T cells (**B**), or in response to ectopic ORP3 in UMUC3 cells (**C**), *n* = 271 (Y235T-shScr), 255 (Y235T-shORP3#1), 264 (Y235T-shORP3#3), 209 (UMUC3-EV), and 216 (UMUC3-ORP3). Experiments show results from three independent sets of experiments pooled together. Mean ± SEM values are shown in the dot plot, and significance was determined by two-tailed Student’s t-test. Before staining, cells were exposed to dimerthylenastron for four hours to increase the proportion of cells in prometaphase. **D–F** Representative pictures showing growth of microtubules in Y235T with shORP3#1 or in UMUC3 cells with ORP3 expression. Measurements of mitotic microtubule plus-end assembly rates in Y235T cells with shORP3 (**E**) or in UMUC3 cells with ectopic ORP3 expression (**F**). *n* = 60 cells are pooled from three independent sets of experiments. Mean ± SEM values are shown in the dot plot, and significance was determined by two-tailed Student’s t test. Scale bars: 5 μm (**A**), and 1 μm (**D**). Images were captured at total magnification of 630 (**A** and **D**)

To further explore the influence of ORP3 on mitosis, we analyzed spindle orientation. Downregulation of ORP3 in Y235T cells leads to spindle pole misplacement out of the cell center during prometaphase. Conversely, ectopic expression of these factors rescues the displacement of spindle poles in UMUC3 cancer cell line (Fig. 2A–C). The quantitation of these events revealed that the spindle pole displacement factor (SPDF) was increased more than twofold upon ORP3 downregulation in Y235T cells compared to Y235T-shScr (0.102 with shScr versus 0.195 with shORP3#1, *P* = 0.0001, and 0.212 with shORP3#3, *P* < 0.0001) (Fig. 2B), while the SPDF was reduced by 40% in UMUC3-ORP3 cells compared to UMUC3-EV control cells (0.441 in UMUC3-EV versus 0.261 UMUC3-ORP3, *P* = 0.0258) (Fig. 2C).

Proper microtubule dynamics is essential for precise spindle orientation and chromosome segregation, and chromosomally instable cancer cells display increased microtubule assembly rates. We explored the influence of ORP3 on microtubule assembly rates via tracking the plus-end movement of EB3 protein which is a common method to observe microtubule dynamics [28] (Fig. 2D–F and Supplementary video files). We find that the knockdown of ORP3 in Y235T cells increases microtubule assembly rates, while its ectopic expression in UMUC3 cells attenuates microtubule assembly rates (Fig. 2D–F). ORP3 knockdown in Y235T improved the microtubule assembly rates by more than twofold compared to Y235T-shScr cells (0.127 μm/s with shScr versus 0.262 μm/s with shORP3#1, *P* = 0.0027, 0.292 μm/s with shORP3#3, *P* = 0.0008) (Fig. 2E), while ectopic expression of ORP3 in UMUC3 cells significantly reduced the microtubule elongation speed (0.263 μm/s in UMUC3-EV vs 0.175 μm/s in UMUC3-ORP3, *P* = 0.0086) (Fig. 2F).

### ORP3 interacts with γ-tubulin during mitosis

**Fig. 3:**
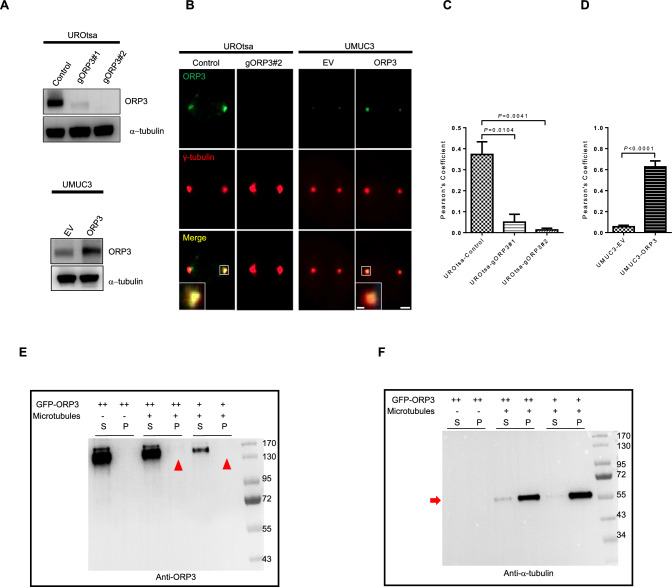
ORP3 interact with γ-tubulin. **A** ORP3 protein level in UROtsa cells with ORP3 knockout (gORP3#1 and #2) as well as in UMUC3 cells with ectopic ORP3 was detected by Western blot. α-tubulin is used as a loading control. *n* = 3 independent experiments were performed. **B** Representative images displaying the colocalization of ORP3 with γ-tubulin during metaphase. Alexa Fluor 488 (green) and Cy5 (red) were used to stain ORP3 and γ-tubulin, respectively. Yellow colour indicates the overlap of ORP3 and γ-tubulin. **C–D** Quantitation of ORP3 and γ-tubulin colocalization in UROtsa (**C**) and UMUC3 (**D**) cells by Pearson’s correlation coefficient**.** *n* = 48 (UROtsa-Control), 43 (UROtsa-gORP3#1), 44 (UROtsa-gORP3#2), 75 (UMUC3-EV), and 70 (UMUC3-ORP3) are pooled from three to four independent experiments. Mean ± SEM values are shown in the bar graph, and significance was determined by two-tailed Student’s t test. **E–F** ORP3 does not bundle microtubules (MT) filaments. 5 × 10^11^ MT/ml and 5–10 μm in length microtubules was incubated with increasing concentrations of ORP3 (relative ORP3 amount is indicated by + or ++). Supernatant (S) and pellet (P) were subjected to 10% SDS-PAGE after high-speed centrifugation at 100,000* g*. (**E**), ORP3, indicated by red arrowheads and (**F**), microtubules, indicated by red arrows, are visualized by specific antibodies. *n* = 3 independent experiments were performed. Scale bars: 2 μm (**B**, main panels) and 0.5 μm (**B**, insets). Images were captured at total magnification of 630 (**B**)

Encouraged by the findings revealing the involvement of ORP3 in microtubules dynamics, we attempted to decipher the underlying mechanisms by which they contribute to these processes during mitosis. To rule out any non-specific or background signals in control cells, we used the CRISPR-Cas9 technology to completely knock-out (KO) ORP3 in UROtsa cells (UROtsa-gORP3#1, #2) (Fig. 3A, B), an SV40-LT transformed ureter-derived cell strain with near-normal karyotype [29]. Of note, repeated attempts to establish KO-Y235T cells or other p53-positive KO-cell lines (e.g. RT4 bladder cancer cell line) failed, likely due to strong apoptotic response caused by endonucleolytic DNA cleavage by the constitutive Cas9 enzyme activity in ureter/bladder cells (unpublished observations). Alternatively, we also used cells with ectopic ORP3 in UMUC3 cells for the colocalization analyzes (Fig. 3A, B). The representative immunofluorescence images and the calculations according to Pearson’s correlation coefficient analysis demonstrate that ORP3 colocalizes with γ-tubulin at the centrosomes both in UROtsa and UMUC3 cells (Fig. 3C, D). In order to evaulate if ORP3 could potentially interact with other subunits of microtubules, we performed the microtubule binding assay. For this purpose, GFP-ORP3 fusion protein was purified from cell extracts (see Materials and Methods for details) and subjected to in vitro microtubule co-sedimentation assays using 100000 g ultracentrifugation. The results of these experiments demonstrate that ORP3 does not directly bind to polymerized microtubules, composed of α- and β-tubulin subunits (Fig. 3E, F). In summary, the colocalization and binding assays indicate that ORP3 colocalizes with γ-tubulin at the centrosomes. These data imply that deregulated expression of ORP3 may have an impact on genome integrity and ploidy-control by compromising the proper functionality of microtubule components.

### The role of ORP3 in bladder cancer

**Fig. 4:**
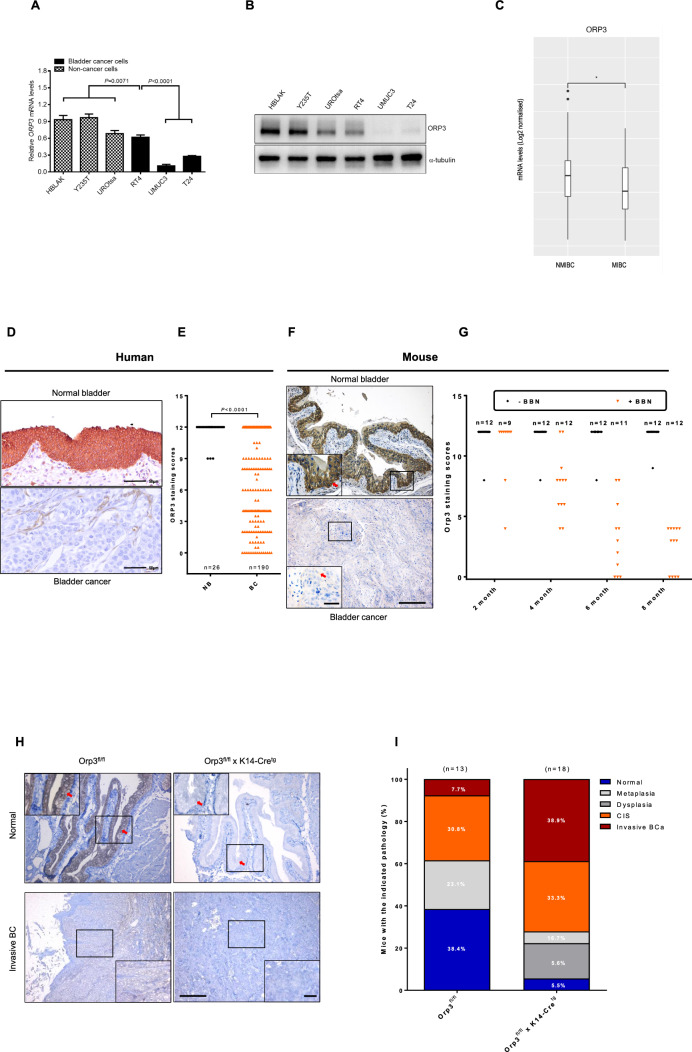
ORP3 as a tumor suppressor in bladder cancer. **A**, **B** ORP3 mRNA and protein expression levels in the indicated cell lines were determined by RT-qPCR (**A**) and Western blot (**B**). The mRNA expression of each gene is calculated in reference to *GAPDH*. Representative results from at least n = 3 independent experiments are depicted. The qPCR shows results of n = 4 technical repeats. Error bars represent Mean ± SEM. α-tubulin is used as protein loading control in Western blot. *n* = 3 independent experiments were performed. **C** Comparison of *ORP3* mRNA levels in the CNUH (GSE13507) cohort of NMIBC (n = 102) and MIBC (n = 63) human bladder tumors. Statistical differences were defined by two-way Fisher’s ANOVA test * = p ≤ 0.05. **D–E** The pictures represented the ORP3 staining in human normal bladder (top) and bladder cancer (bottom) tissues (**D**) immunohistochemistry, and the quantitation of ORP3 staining (**E**) performed by immunohistochemistry in the patient cohort of NB (n = 26) and BC (n = 190). Mean ± SEM values are shown in the bar graph, and significance was determined by Welch’s test with 95^%^ confidence interval. NB, normal bladder; BC, bladder cancer. **F–G** The pictures indicated the Orp3 staining in mouse normal bladder (top) and bladder cancer (bottom) tissues (**F**), and the quantitation of Orp3 (**G**) staining in normal bladder and cancer bladder tissues during the BBN-induced BC progression in mice. Red arrows indicate Orp3 staining. In contrast to the control mice (black dots), which were fed water, the expression of Orp3 gradually decreased over the course of the BBN-treatment (orange dots). Mean ± SEM values are shown in the bar graph, and significance was determined by two-tailed Student’s t test. **H** The representative images of Orp3 staining in Orp3-proficient mice (Orp3^fl/fl^ x K14-Cre^tg^) and Orp3 knockout (Orp3^fl/fl^ x K14-Cre^tg^). Red arrows indicate Orp3. **I** The bar graph shows the percentage of different pathologic stages of bladder in mouse. Scale bars: 200 μm (**F**, **H** main panels) and 50 μm (**F**, **H**, insets, **D**). Images were captured at total magnification of 400× (**D**), 100× (**F, H**, main panels), and 630× (**F**, **H**, insets)

Based on the high expression levels of ORP3 in urothelial cells and its involvement in ploidy-control, we assessed RNA and protein expression levels of ORP3 during BC progression. Firstly, mRNA and protein levels of ORP3 were determined in three BC cell lines (RT4, T24 and UMUC3) and three immortalized ureter epithelial cells (HBLAK, Y235T and UROtsa). Notably, mRNA and protein levels were higher in immortalized cells compared to BC cell lines (Fig. 4A, B). Interestingly, ORP3 expression was moderate in the RT4 BC cell line, which is known to have low invasive capacity compared to the T24 and UMUC3 BC cell lines with high invasive capacities. Along with the tissue expression data (Fig. 1A, B), the results suggest that ORP3 expression is reduced in BC.

To investigate the association of ORP3 with bladder cancer progression, we next compared its mRNA levels using CNUH dataset cohorts (GSE13507) of balanced NMIBC (n = 102) versus MIBC (n = 63) human bladder tumors [30]. We observed statistically significant differences showing reduced *ORP3* expression in MIBC compared to NMIBC (Fig. 4C). In addition, we determined *ORP3* expression by immunohistochemistry (IHC) on human and mouse BC samples. IHC staining for the detection of ORP3 on tissue microarrays (TMA), comprised of 190 human MIBC, along with 26 apparently normal bladder tissue samples were performed at the University of Erlangen-Nuremberg. We observed decreased ORP3 expression in human MIBC, compared to normal bladder (Fig. 4D; see Supplementary Fig. 2 for more details on MIBC cohort). We also found reduced ORP3 expression throughout tumor progression in the N-butyl-N-(4-hydroxybutyl)-nitrosamine (BBN)-induced mouse BC model (Fig. 4F, G and Supplementary Fig. 3). The BBN-induced mouse BC mimics genetic and histological features of human BC and thus is a promising animal model for muscle invasive bladder cancer [15, 31]. In our study, early genetic and histological changes were noticed at 6–8 weeks post-treatment and bladder metaplasia was detectable in male and female mice with BBN treatment (+ BBN) after 4 months post-treatment. Notably, tumors were increasingly evident from the fourth month of BBN-treatment (Supplementary Fig. 3A, 3B). As expected, mice in the control group without BBN-treatment (–BBN) did not develop tumors within the observation period (up to 8 months).

Finally, we used a tissue-specific Orp3 knockout mouse model to investigate the role of Orp3 in bladder cancer progression (see Materials and Methods for details). It was previously demonstrated that a keratin 14 expressing subpopulation of urothelial cells represents cells of origin of urothelial cancer [32]. Therefore, we generated bi-transgenic mice to knockout Orp3 in mouse bladder epithelial cells using the keratin 14-Cre (K14-Cre) mouse model. Since mice with the tissue-specific Orp3-KO (Orp3^fl/fl^ × K14-Cre^tg^) did not develop tumors over a period of 2 years (not shown), we used the above described BBN-induced BC progression model to elucidate potential contribution of Orp3 to BC progression. In this case, tumor development was monitored for a maximum of 4 months. IHC confirmed the loss of Orp3 expression in the knockout mice (Fig. 4H). The evaluation of the histological analyzes revealed a fivefold increase in the incidence of BBN-induced invasive bladder carcinoma in Orp3-KO mice (Orp3^fl/fl^ × K14-Cre^tg^) compared to control mice (Orp3^fl/fl^) (38.9% in Orp3^fl/fl^ × K14-Cre^tg^ versus and 7.7% in Orp3^fl/fl^ mice) (Fig. 4I). The observation that the bladder tissue was normal in more than 1/3 of Orp3-proficient mice (5.5% in Orp3-KO and 38.4% in Orp3-proficient control group) indicates that Orp3 may have a tumor suppressor role, which is in line with our previous report showing that the conventional Orp3-KO mice developed B-cell lymphoma [17]. However, there was no significant difference in the incidence of carcinoma in situ (CIS) (33.3% in Orp3-KO and 30.8% in control group), suggesting that Orp3 impaired the progression of tumors towards invasive carcinoma. Notably, invasive tumors typically appeared earlier in the Orp3-KO mice (not shown). In summary, the Orp3-KO mice studies suggest a role of Orp3 in tumor initiation and progression. Altogether, these data showed downregulation of ORP3 in cells and tissues with higher invasive features and during progression of bladder cancer.

### ORP3 impairs invasion and migration capacities of BC cells

**Fig. 5:**
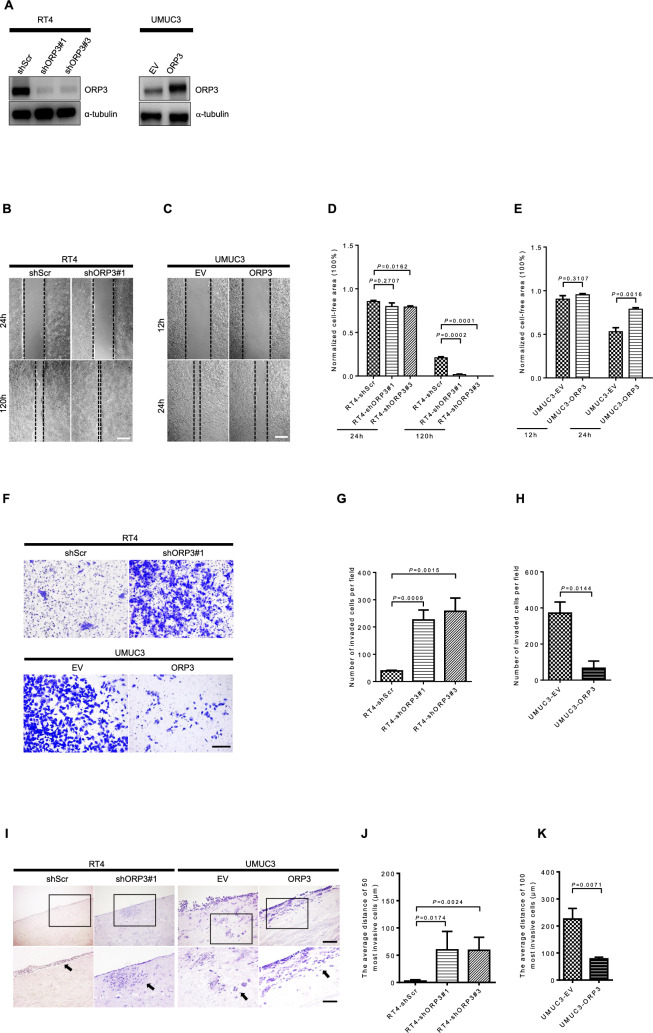
ORP3 inhibits cells invasion and migration. **A** Western blot shows protein levels of ORP3 in RT4 cells that were infected with shRNAs targeting their expression, and in UMUC3 cells with ectopic ORP3 expression. The expression of α-tubulin served as the loading control. The Western blots were repeated for three times. **B**, **C** Wound healing/scratch assay demonstrating the impact of ORP3 on the migratory capacity of BC cell lines. The representative pictures, captured at 24 and 120 h in case of RT4 cells and at 12 and 24 h in case of UMUC3 cells, show that the knockdown of ORP3 improves the migratory capacity of RT4 cells while the ectopic expression impairs the migratory capacity of UMUC3 cells, respectively. **D**, **E**, Quantitation of normalized cell free area of RT4 cells expressing the indicated shRNAs targeting ORP3 (**D**) or UMUC3 cells with ectopic ORP3 expression (**E**). *n* = 3 independent experiments were performed. Mean ± SEM values are shown in the bar graphs, and significance was determined by two-tailed Student’s t test. **F** Boyden chamber approach demonstrating the impact of ORP3 on the invasion capacity of BC cell lines. The representative pictures showing cells invaded through the Boyden chamber, stained at 144 h post-seeding in case of RT4 cells (top) and at 48 h post-seeding in case of UMUC3 cells (bottom), demonstrate that the knockdown of ORP3 improves the invasive capacity of RT4 cells while the ectopic expression impairs the invasive capacity of UMUC3 cells, respectively. **G, H** Quantitation of invasive capacity of RT4 cells expressing the indicated shRNAs targeting ORP3 (**G**) or UMUC3 cells with ectopic ORP3 expression (**H**). *n* = 3 independent experiments were performed. Mean ± SEM values are shown in the bar graphs, and significance was determined by two-tailed Student’s t test. **I, K** Porcine bladder ex vivo organ culture approach demonstrating the impact of ORP3 on the invasion capacity of BC cell lines (see Supplemental Fig. 5 for measuring and quantification of the invasive capacity of BC cells in the porcine bladder ex vivo organ culture model). Representative images of H&E staining. The cells were seeded on the surface of the de-epithelized porcine bladder for 21 days (RT4), or 14 days (UMUC3), respectively. The results of the ex vivo organ culture model demonstrate that the knockdown of ORP3 improves the invasive capacity of RT4 cells while their ectopic expression impairs the invasive capacity of UMUC3 cells, respectively. Insets: enlarged images of the areas shown by black boxes. Black arrows point to the cells that have spread the farthest. The impact of ORP3 on the invasive capacity of BC cell lines was calculated as above. *n* = 4 (RT4-shScr), *n* = 3 (RT4-shORP3#1), *n* = 5 (RT4-shORP3#3), and *n* = 3 (UMUC3-EV), *n* = 4 (UMUC3-ORP3) independent experiments were performed. Mean ± SEM values are shown in the bar graph, and significance was determined by two-tailed Student’s t-test. Scale bars: 200 μm (**B**, **C**, **F**), 200 μm (**I**, main panels) and 100 μm (**I**, insets). Images were captured at total magnification of 50× (**B, C**), 100× (**F**), 100× (**I**, main panels), and 200 × (**I**, insets)

In order to obtain further insight into the function of ORP3 in tumor progression, its expression was knocked down in RT4, a BC cell line with low invasive and migratory capacities, as well as ectopically expressed in UMUC3, a BC cell line with high invasive and migratory capacities. Efficient knockdown and ectopic expression were confirmed by Western blot (Fig. 5A). First, the impact of ORP3 on the migratory capacity of cells was investigated by the wound healing assay. The results of these experiments clearly show that the reduced expression of ORP3 results in an improved migration capacity of the RT4 cells, while ORP3 ectopic expression impaired the migration capacity of UMUC3 cells (Fig. 5B–E). Since we did not detect changes in cell counts in response to altered ORP3 expression, we conclude that the impact of these factors on the migratory capacity of cells was genuine and not due to changes in cell proliferation (Supplementary Fig. 4). Please note that distinct timeframes were employed for RT4 and UMUC3 cells due to their differing migration capacities. We have next applied the Boyden chamber approach to address the impact of ORP3 on cell invasion and migration. The representative pictures and the quantitation of these experiments clearly show that the knockdown of ORP3 improves the migratory and invasive capacities of the RT4 cell lines in culture, while its ectopic expression impairs the migratory and invasive capacities of the UMUC3 cell line (Fig. 5F–H). In addition to the in vitro Boyden chamber experiment, the ex vivo porcine organ culture method was used to further measure the impact of ORP3 on the invasive capacities of RT4 and UMUC3 cells (Figs. 5I–K). As displayed in representative pictures, RT4-shScr control cells formed an epithelium-like layer on the surface of the de-epithelized porcine bladder tissue, while RT4 cells with reduced ORP3 expression invaded into the stroma and muscle of the porcine tissue (Fig. 5I). By measuring the distance from the tissue surface to the invasive front (see Supplementary Fig. 5 for the measurement), we were able to quantify the effect of these factors on tissue invasion. While ORP3 knockdown improved the invasion ability of RT4 cells by about 24-fold (2.5 µm with RT4-shScr versus 59.8 µm with RT4-shORP3#1, P = 0.0174, and 58.9 µm with RT4-shORP3#2, P = 0.0024), ectopic expression of ORP3 decreased the ability of UMUC3 cells to invade into the tissue stroma/muscle by more than 65% in comparison to UMUC3-EV (225.7 µm with UMUC3-EV vs 78.1 µm with UMUC3-ORP3, P = 0.0071) (Fig. 5J, K).

### ORP3 regulates invadopodia formation via actin dynamics

**Fig. 6:**
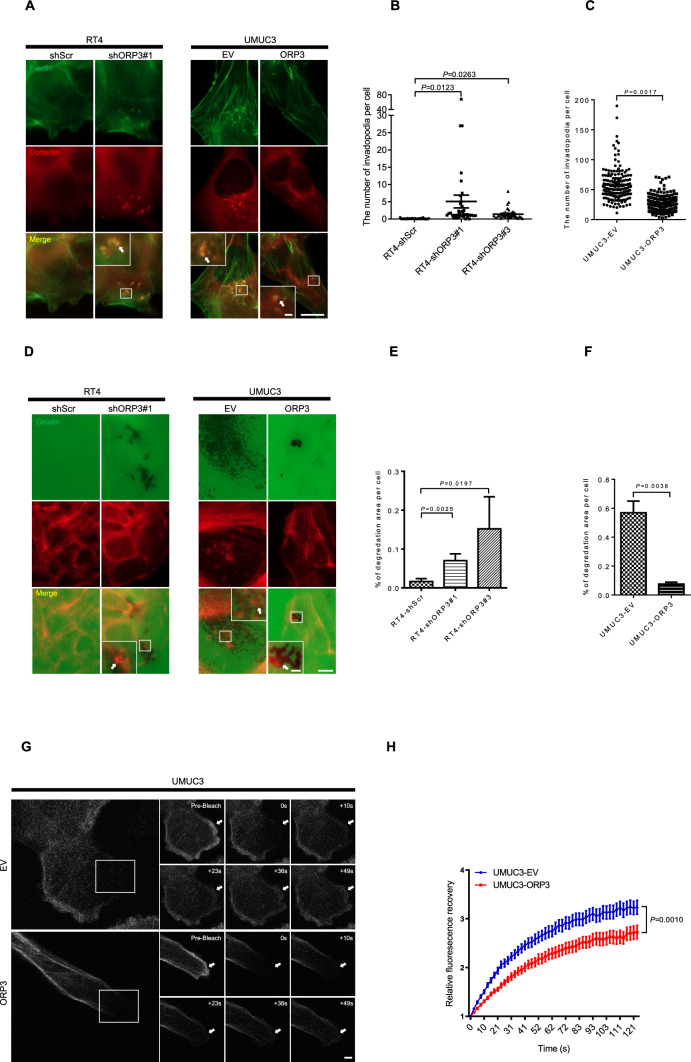
ORP3 regulates invadopodia formation via actin dynamics.** A** Representative images demonstrating invadopodia formation of RT4 cells upon ORP3 knockdown with the indicated shRNAs or UMUC3 cells upon ectopic ORP3 expression, respectively. **B**, **C** Quantitation of the invadopodia number was performed as above. *n* = 936 (RT4-shScr), *n* = 513 (RT4-shORP3#1), *n* = 578 (RT4-shORP3#3), and *n* = 171 (UMUC3-EV), *n* = 179 (UMUC3-ORP3). Cells are pooled from three independent sets of experiments. Alexa Fluor 488-phalloidin (green) and Cy5 (red) stain F-actin or cortactin, respectively. The invadopodia structures are characterized by yellow dots that display F-actin and cortactin colocalization. Insets: enlarged images of the areas shown by white boxes. The invadopodia are marked with white arrows. Mean ± SEM values are shown in the dot plot, and significance was determined by two-tailed Student’s t test. **D** Example pictures show the gelatin degradation in RT4 cells upon ORP3 knockdown with the indicated shRNAs or UMUC3 cells upon ectopic ORP3 expression, respectively. **E**, **F** Quantitation of the gelatin degradation capacity of the cells was performed by measuring the degradation area per cell. *n* = 9767 (RT4-shScr), *n* = 8125 (RT4-shORP3#1), *n* = 5633 (RT4-shORP3#3), *n* = 1588 (UMUC3-EV), and *n* = 1067 (UMUC3-ORP3) are pooled from three to four independent experiments. Mean ± SEM values are shown in the bar graph, and significance was determined by two-tailed Student’s t test.** G** Representative images show the LifeAct–Ruby signal recovery time after bleaching the LifeAct–Ruby signal in UMUC3 cells with or without ORP3.** H** The quantitation in UMUC3 cells with or without ORP3. *n* = 29 (UMUC3-EV) and 31 (UMUC3-ORP3) cells are pooled from three independent sets of experiments. The data points in the graphs represent mean ± SEM. *P* values were determined at t = 49 s by two-tailed Student’s t test. Scale bars: 10 μm (**A**, **D** main panels), 1 μm (**A**, insets), 2 μm (**D**, insets) and 1 μm (**G**). Images were captured at total magnification of 630× (**A, G**) 400× (**D**)

The above findings demonstrate that ORP3 influence tumor cell migration and invasion capacities. In conclusion, their altered expression or malfunction may have an impact on the metastatic spread of cancer. Invadopodia play an important role in cell attachment and remodeling of the extracellular matrix (ECM) during these processes. Therefore, we investigated whether ORP3 influenced the formation of invadopodia employing a Cortactin and F-actin colocalization approach (Fig. 6A–C). ORP3 knockdown in RT4 cells improved invadopodia formation by 13–25 fold compared to RT4-shScr cells (mean values: 0.101 with RT4-shScr versus 2.562 with RT4-shORP3#1, *P* = 0.0085, and 1.365 with RT4-shORP3#3, *P* = 0.0323), while ectopic ORP3 expression in UMUC3 cells impaired their invadopodia formation ability by about 50% compared to the UMUC3-EV cells (mean values: 57.310 with UMUC3-EV vs 26.084 with UMUC3-ORP3, *P* = 0.0017). In addition, we conducted gelatin degradation experiments to examine the impact of ORP3 on invadopodia formation at the subcellular level. Knockdown of ORP3 significantly increased gelatin degradation activity of RT4 cells, visible as black areas both at the outside edges and more impressively at the inside of cell islands. Conversely, ectopic expression of ORP3 in UMUC3 cells resulted in a 85% reduction of gelatin degradation activity, respectively, compared with UMUC3 control cells containing an empty vector (Fig. 6D–F).

Regulation of actin dynamics is a crucial process for cell migration, invadopodia formation and cell invasion. Therefore, we explored the influence of ORP3 on peripheral actin dynamics by a fluorescence recovery after photobleaching (FRAP) approach. This assay is based on measuring the fluorescence recovery of the F-actin-binding factor LifeAct- Ruby to visualize actin polymerization. Compared to UMUC3-EV cells, relative fluorescence recovery was impaired in UMUC3-ORP3 after photobleaching the LifeAct- Ruby signal located in lamellipodia (Fig. 6G, H). ORP3 overexpression weakened the relative fluorescence signal by more than 15% at both 49 s and 126 s (2.582 at 49 s and 3.235 at 126 s with EV versus 2.111 at 49 s and 2.727 at 126 s with ectopic ORP3,* P* = 0.0010). These results indicate that ORP3 is involved in the assembly process of actin helices.

### ORP3 binds directly to actin filaments

**Fig. 7:**
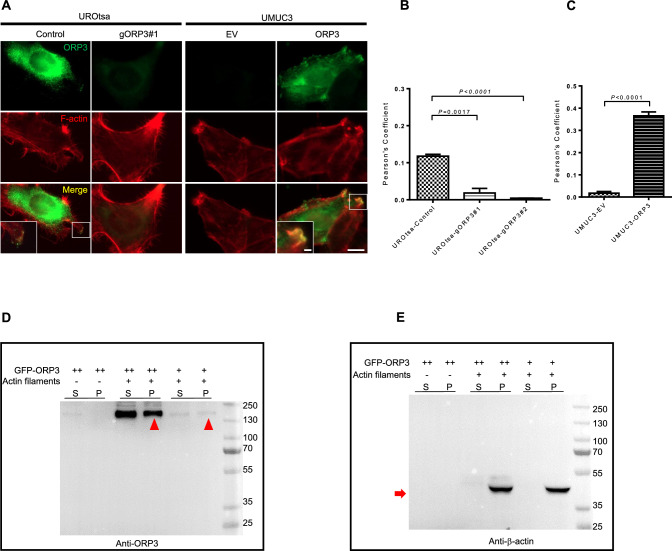
ORP3 interacts with actin filaments. The knockout of ORP3 in UROtsa cells using the CRISPR-cas9 technology was described in the main text above. **A** Representative images displaying the colocalization of ORP3 with F-actin in UROtsa and UMUC3 cells. **B**, **C** Quantitation of ORP3 and F-actin colocalization in UROtsa (**B**) and UMUC3 (**C**) cells by Pearson’s correlation coefficient. *n* = 233 (UROtsa-Control), *n* = 228 (UROtsa-gORP3#1), *n* = 226 (UROtsa-gORP3#2), *n* = 475 (UMUC3-EV), and *n* = 704 (UMUC3-ORP3) are pooled from three independent experiments. Mean ± SEM values are shown in the bar graph, and significance was determined by two-tailed Student’s t test. The F-actin is stained by Alexa Fluor 647-phalloidin (red). ORP3 are stained by Alexa Fluor 488 (green). The overlap of ORP3 with F-actin is indicated with yellow colour. Insets: enlarged images of the colocalized areas shown by white boxes. **D**, **E** ORP3 bundle actin filaments in a dose-dependent manner. Actin (2.5 mg/ml) concentrations of ORP3 (relative ORP3 amount is indicated by + or ++). Supernatant (S) and pellet (P) were subjected to 10% SDS-PAGE after high-speed centrifugation at 100,000* g*. (**D**), ORP3, indicated by red arrowheads, (**E**), and actin filaments, indicated by red arrows are visualized by specific antibodies. *n* = 3 independent experiments were performed. Scale bars:10 μm (**A**, main panels) and 2 μm (**A**, insets). Images were captured at total magnification of 630 × (**A**)

Based on the above data showing a contribution of ORP3 in invadopodia formation and actin dynamics, we examined a potential colocalization of ORP3 with F-actin, the main component for cell protrusions. For this purpose, we used the UROtsa-gORP3 knockout cells, as introduced and described in Fig. 3, as well as UMUC3 cells with ectopic overexpression of ORP3. As shown by the co-immunofluorescence staining results, ORP3 signals overlapped with F-actin at the lamellipodia area of UROtsa control cells and UMUC3 cells with ectopic overexpression of ORP3, while there was no overlap in UROtsa-gORP3 cells or UMUC3-EV control cells (Fig. 7A–C). The quantitation of the Pearson’s correlation coefficient (PCC) showed that ORP3 knockout in UROtsa cells reduced the PCC by about 85% with UROtsa-gORP3#1 and by more than 95% with UROtsa-gORP3#2 (0.118 in control vs 0.018 in UROtsa-gORP3#1, *P* = 0.0017, and 0.004 in UROtsa-gORP3#2, *P* < 0.0001) (Fig. 7B). The PCC in UMUC3 cells with ORP3 overexpression was 20-fold higher than in control cells (0.017 with empty vector versus 0.365 with ORP3 overexpression,* P* < 0.0001) (Fig. 7C). To further investigate a potential interaction of ORP3 with F-actin, co-sedimentation assays were performed. As shown by the Western blot experiments, ORP3 protein can directly bind to polymerized actin in a dose-dependent manner (Fig. 7D, E).

## Discussion

In this study, we provide novel insights into the molecular mechanisms that reveal how ORP3 contributes to genome stability, how ORP3 promotes the initiation and progression of bladder cancer, and how ORP3 impacts the migration and invasion capabilities of bladder cancer cells. ORP3, has well-defined roles in the lipid trafficking and cell signaling [33]. Importantly, ORP3 was found to play important role in the dynamic of phosphatidylinositol 4-phosphate and Ca2+ , which influence several biological processes, including cell migration and proliferation [10]. Notably, ORP3 has been shown to influence adhesion, migration and spreading of cancer cells [8]. Using a genome-wide iRNA screening, we have recently revealed an unprecedented engagement of ORP3 in ploidy-control, since ORP3 deficiency resulted in aneuploidy and improved genome instability. Moreover, loss of ORP3 promoted malignant cell transformation and tumorigenesis [17]. However, the molecular mechanims how ORP3 is linked to genome stability and how the loss of ORP3 promotes cancer are still being investigated. It also remains to be explored whether the two functions that are associated with ORP3, namely gonome stability and cell adhesion/spreading, are mediated by the same molecular mechanisms.

Lehto et al. demonstrated in a seminal work on the ORP gene family that the 12 human ORPs display unique tissue-specific expression patterns [2]. Even though this study covered an array of tissues, the expression of ORPs in a number of tissues was not determined. In the present study, we assessed the mRNA levels of human *ORP3* in 20 human tissues from which total RNA was commercially accessible. Interestingly, strong ORP3 expression was found in the epithelial cells of the ureter and bladder, both at RNA and protein levels. We thus investigated the potential roles of ORP3 in bladder, as aneuploidy is positively correlated with the invasive potential of bladder cancer (BC), with muscle-invasive BC (MIBC) being highly aneuploid compared to the non-muscle-invasive BC (NMIBC). Of note, increased aneuploidy in MIBC is frequently associated with progression to metastatic disease and poor prognosis [34]. Importantly, MIBC is associated with higher incidence of distant metastasis compared with NMIBC [35–37].

We find that ORP3 interacts with γ-tubulin during cell division and is associated with components of actin cytoskeleton. ORP3 deficiency impairs correct chromosomal segregation, resulting in aneuploidy and genomic instability in ureter-derived, telomerase-immortalized Y235T cells with otherwise normal karyotype. Impairment of γ-tubulin-ORP3 interaction is associated with increased microtubule dynamic rates. ORP3 downregulation also results in increased rates of multipolar spindles, defective spindle pole location, likely contributing to defects in chromosome segregation and leading to the generation of lagging chromosomes and aneuploidy [28, 38–40]. On the other hand, deregulated actin cytoskeleton dynamics in response to altered ORP3 levels has an impact on cell motility and influences migration and invasive behaviour of BC cells. ORP3 knockdown in the non-invasive BC cell line RT4 or ectopic ORP3 in invasive BC cell lines (T24/UMUC3) clearly support a role of ORP3 in cell migration and invasion. The genuine role of ORP3 as a tumor suppressor factor is also indicated by reduced ORP3 levels in invasive BC cell lines and during the progression of BC in humans and mice. Notably, BBN-treated Orp3-KO mice develop earlier and more invasive carcinoma compared to BBN-treated Orp3-proficient mice.

We find it worth noting that ORP3 affects both cell invasion and migration as well as genome stability through interactions with cytoskeletal components. We show that loosened interactions of ORP3 with γ-tubulin impairs proper chromosome segregation during cell division while altered ORP3 levels influence the dynamics of F-actin. Both have consequences on cell division and cell motility. The invasive capacity of tumor cells is characterized by loss of cell–cell junctions and adhesion, increased motility, as well as by rearrangements of cytoskeletal proteins [41–43]. A large number of effector and regulatory factors are involved in the process of cell migration and invasion [44]. Though generally considered a purely actin-based process, microtubules also have crucial roles in cell migration [45–47]. In fact, actin and microtubules cooperate both in cell division and cell motility [48–50]. It was also demonstrated that actin-microtubule interplay coordinates spindle assembly in human oocytes [51]. Microtubules have essential roles in cell division as they search for and capture chromosomes in mitosis [52, 53]. While the relevance of deregulated microtubule machinery in genome instability is thoroughly studied and is undeniable, the impact of actin-dynamics in genome stability is less-well studied. It was demonstrated that the actinomyosin cortex plays an eminent role in mitotic rounding in spindle alignment [54]. The intact actin cytoskeleton for proper spindle positioning is crucial as reflected in the requirement for a number of actin regulators including ERM (ezrin, radixin and moesin) family members [55], cdc42 [56] and focal adhesion molecules [57]. In this line, it was shown that the expression of a constitutively active mutant of the Wiskott-Aldrich Syndrome protein (WASP) leads to abnormally high levels of F-actin through deregulated activation of the Arp2/3 complex and causes aneuploidy in cells [58, 59]. In this line, we have recently shown that the knockdown of TKS5 (SH3PXD2A), a well-known regulator of invadopodia formation and cell migration, influences F-actin-cortactin colocalization and induces aneuploidy in human ureter-derived epithelial cells [23]. Other well-studied regulators of actin-dynamics, the RhoGTPases (Ras homologous family GTPases), are also known to be involved in local dynamics of microtubules, centrosome dynamics and control of proper chromosome segregation [60, 61]. Notably, deregulated expression and/or activation of Rho GTPases often correlates with tumor progression, metastasis, and poor prognosis [62].

These findings may also provide an explanation for recent observations, which revealed that a large number of genes and pathways can influence genome integrity and ploidy-control. Several laboratories identified a growing list of chemicals, genetic factors (including oncogenes), and cellular stress factors that are implicated in the induction of aneuploidy in eukaryotic cells [63, 64]. Supported by iRNA screens on focus set libraries, Conery and Harlow suggested that the number of aneuploidy inducing gene mutations may be much higher than previously thought involving a variety of cellular signaling pathways [65]. This conclusion is consistent with our previous findings from a genome-wide iRNA screening, in which we identified a large set of cancer-associated genes with a novel role in aneuploidy induction [17]. It is reasonable to assume that a dysfunction of other factors that are structurally associated to cytoskeletal components or that influence cell migration/invasion through actin and/or microtubule dynamics would results in aneuploidy.

### Supplementary Information

Below is the link to the electronic supplementary material.

Supplementary file1 (PDF 809 KB)

Supplementary file2 (AVI 435 KB)

Supplementary file3 (AVI 445 KB)

Supplementary file4 (AVI 310 KB)

Supplementary file5 (AVI 316 KB)

## Data Availability

All data analysed during this study to evaluate the conclusions are included within the article or available in supplemental information. Additional related data need to be requested from the corresponding author.
