# Supplementary material for: Loss of ORP3 induces aneuploidy and promotes bladder cancer cell invasion through deregulated microtubule and actin dynamics
Source: Cell Mol Life Sci. 2023 Sep 22;80(10):299. doi: 10.1007/s00018-023-04959-6 (PMC10516806; doi:10.1007/s00018-023-04959-6)
Supplement: Supplementary file 1 — Supplementary file1 (PDF 809 KB) [file 18_2023_4959_MOESM1_ESM.pdf]

Supplementary Figure 1

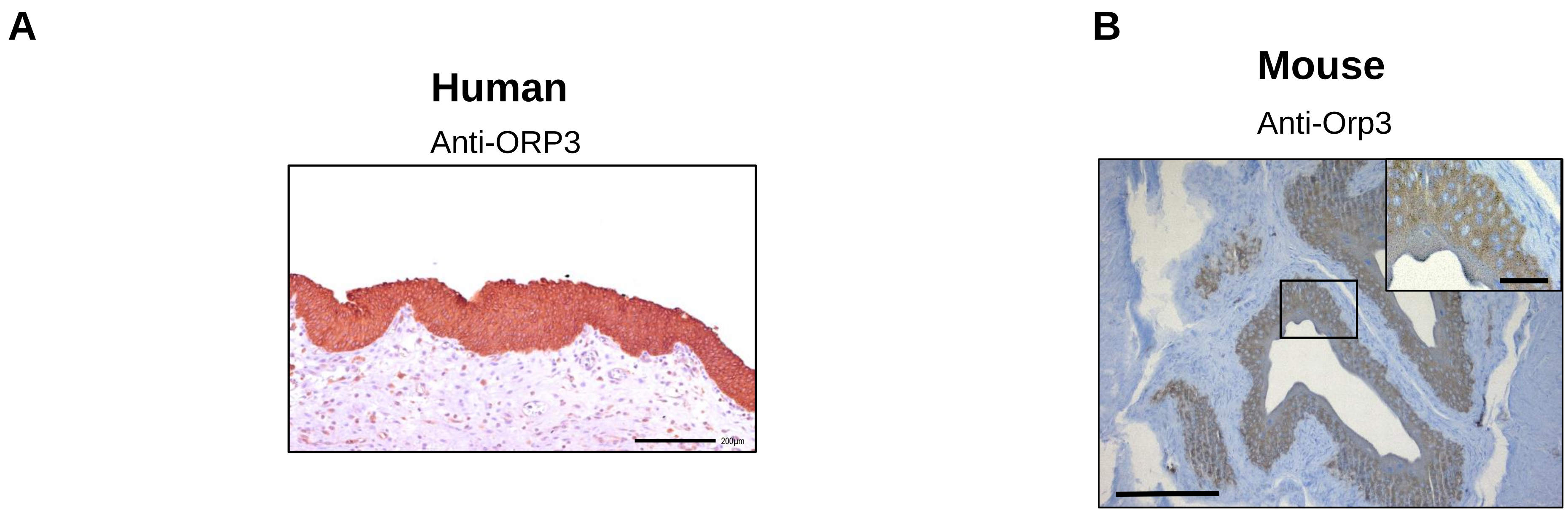

Supplementary Figure 1. Detection of ORP3 by IHC in human and mouse normal bladder tissues.

A IHC of ORP3 in normal human bladder tissue demonstrates strong expression in the epithelium, ranging from basal cells to ambrella cells. Please note that the ORP3 image was captured from the same staining as shown in Fig. 4D. Here, the image was captured at total magnification of 200.

B IHC of ORP3 in normal mouse bladder tissue demonstrates strong expression in the epithelium, ranging from basal cells to ambrella cells. Images were captured at total magnification of 100 (main panels) and 630 (insets).

Supplementary Figure 2

A

**ORP3**	
Total	190
**OS**	
dead	140
live	50
**RFS**	
Recurrence	107
No recurrence	83
**pT stage**	
pT2	49
pT3	95
pT4	46
**pN stage**	
pN0	120
pN1	16
pN2	38
pNX	16
**WHO grading**	
high grade	190
low grade	0
**Gender**	
Male	136
Female	54
**Age**	
37-59	35
60-79	119
80-91	36
**Histology types**	
Neuroendocrine	7
Urothelial	183
**MDACC subtype**	
Basal	84
DN	10
Luminal	96
Unkown	0
**Variant Histology**	
Clear Cell	1
Glandular	3
Glycogen rich	0
LNUC	1
Lymphoepithelioma-like	2
MPUC	10
Nested	2
Neuroendocrine	9
NOS	101
NUC	0
PUC	7
Rhabdoid	2
Sarcomatoid	10
Squamous	40
Trophoblastoid	2

B

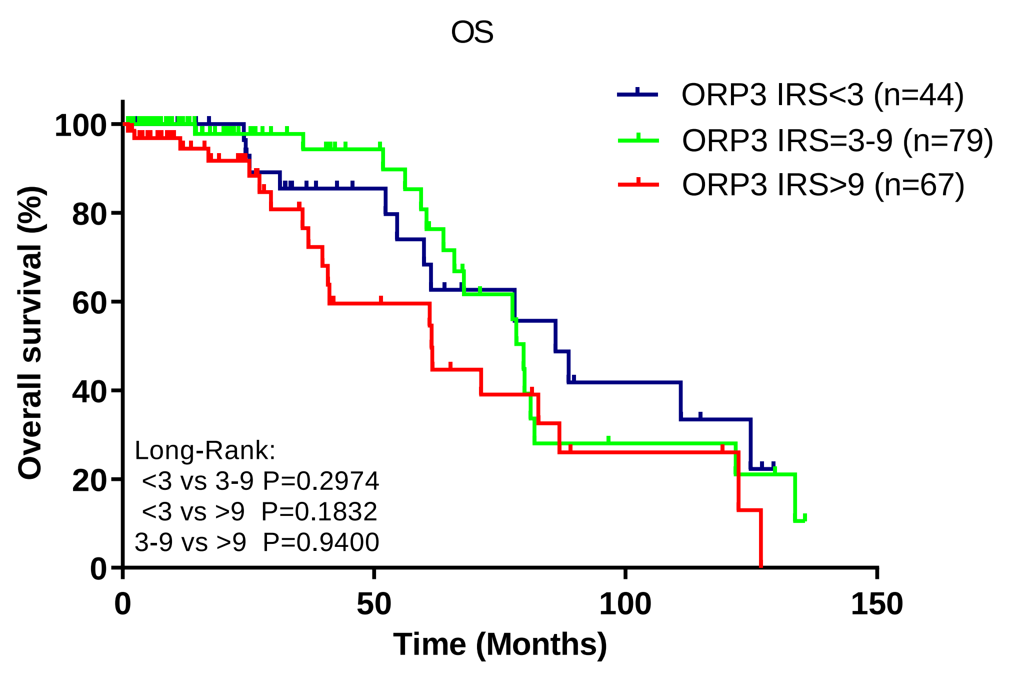

C

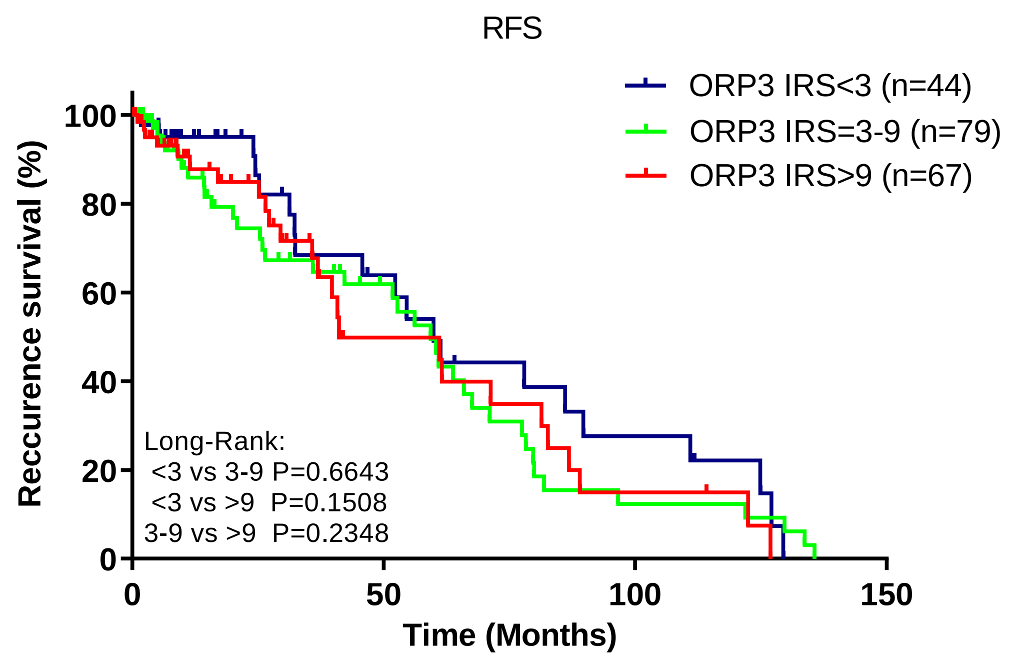

Supplementary Figure 2. Clinical data of patients with BC and the evaluation of patient survival.

A The details of clinical data are shown in the excel.

B, C The overall survival (OS) and recurrence-free survival (RFS) of ORP3 of patients with MIBC. The groups are divided according to staining intensities: IRS<3 (n =44), IRS=3-9 (n =79), and IRS>9 (n =67).

Supplementary Figure 3

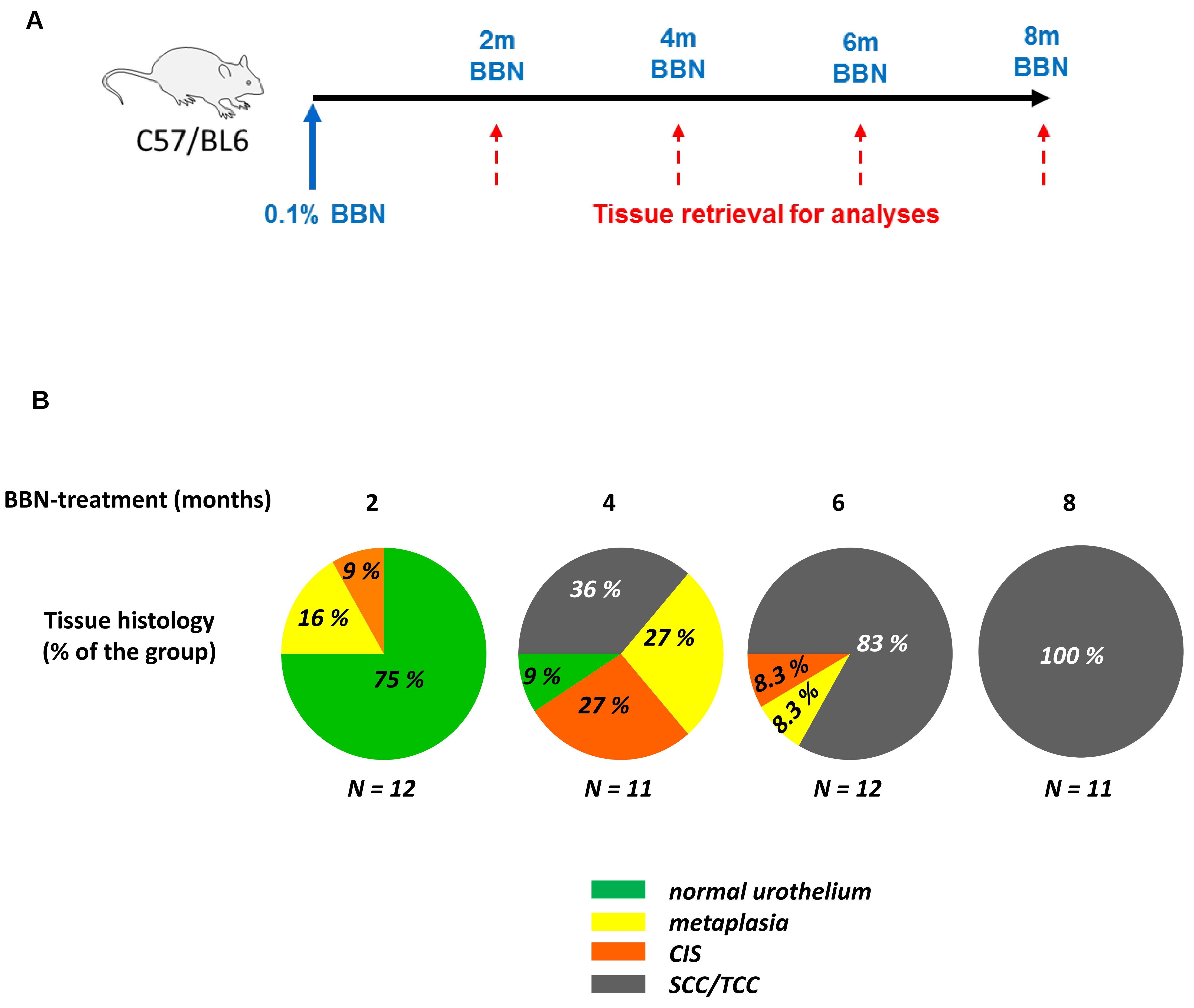

Supplementary Figure 3. N-butyl-N-(4-hydroxybutyl) nitrosamine (BBN)-induced BC progression in mice.

A The flow chart shows the experimental setup. Male and female C57/BL6 mice were fed with 0.1% N-butyl-N-(4-hydroxybutyl) nitrosamine (BBN) in water, starting at the age of 8-10 weeks. The mice were sacrificed and the bladder tissues were collected after 2, 4, 6, and 8 months' treatment with BBN.

B The pie charts show the results of histopathological evaluation of mice bladder tissues at the indicated time-points.

Supplementary Figure 4

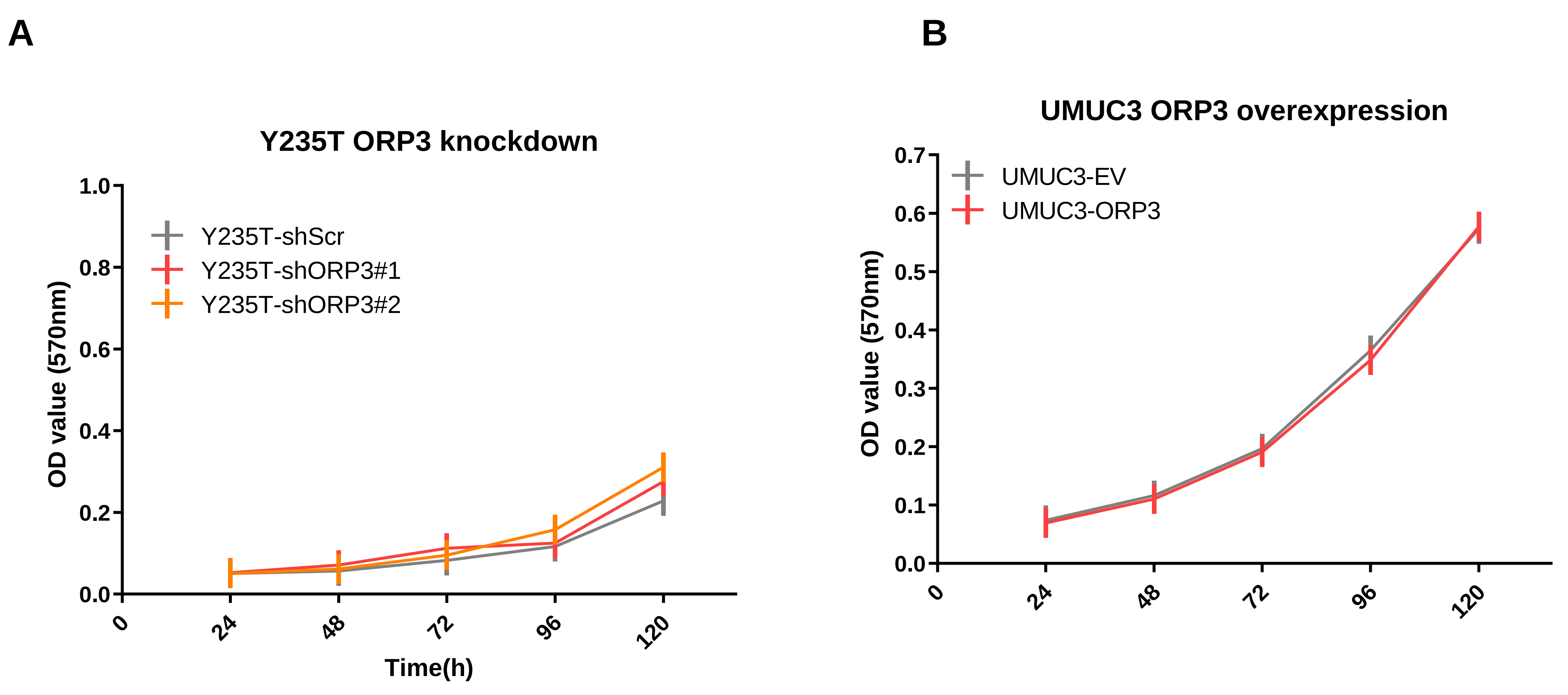

Supplementary Figure 4. ORP3 has no impact on cell viability and proliferation.

A The MTT assay shows that the knockdown of ORP3 in Y235T cells did not impact cell survival and proliferation.

B The MTT assay shows that the overexpression of ORP3 and in UMUC3 cells has no influence of cell viability.

Supplementary Figure 5

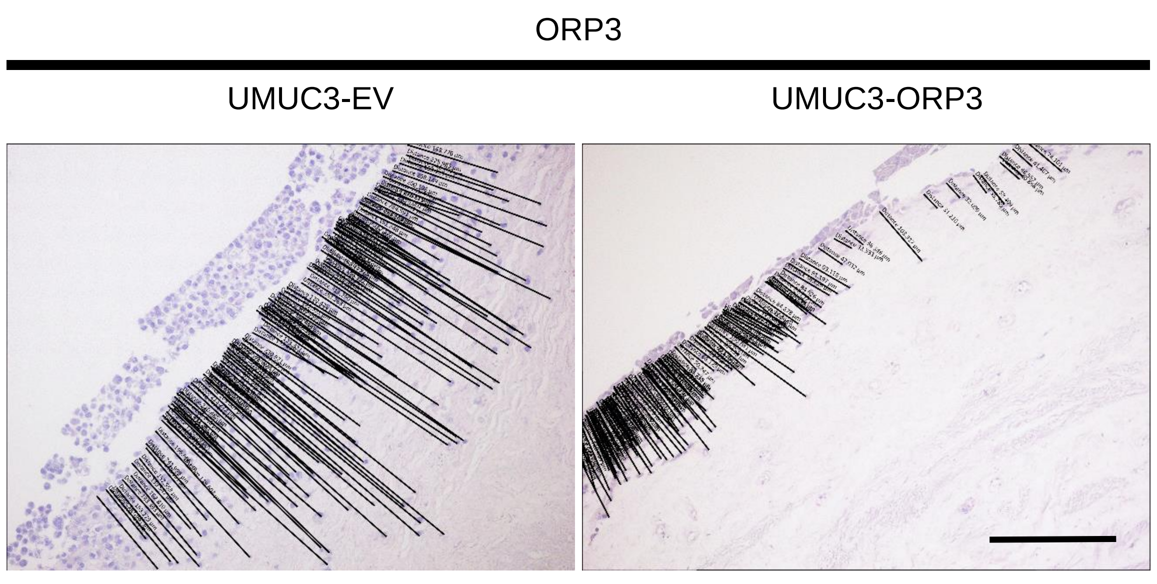

Supplementary Figure 5. Example figure showing the measurement of the invasive capacity of cells in the *ex vivo* porcine bladder organ model.
The representative pictures show how the invasive depth is measured by ZEN software. Pictures obtained under 100× magnification using a Zeiss TCS SP5 confocal microscope at random from 6 areas of bladder tissues were used to quantify the amount of cell invasion. In the example pictures (a, b), ZEN software was used to quantify the distance between the tissue-invaded cells and the closest surface for the 100 deepest invasive UMUC3 cells (UMUC3-EV, and UMUC3-ORP3, respectively), and the mean distance was determined. Scale bars: 200µm.

Suppl. Tables

Table I: Cloning primers

**Target gene**	Direction	Sequence 5’ - 3’
**For the cloning of GFP-EB3 in pBABE-Bla**	Forward	TCAGCTGAATTCTCATCTGCCACCATGGCCGTCAATGT
	Reverse	TCAGCTGTCGACCCTCGCTTTACTTGTACAGCTCGTCC

Table II: shRNA vectors and sequences

**Plasmid**	**Target Sequence**	**Selection marker**	**Other**
pGIPZ shScramble	CCCGCCTGAAGTCTCTGATTAA	Puromycin	GFP
pGIPZ shORP3#a	CGGCACAAACCTGTGTTTATA	Puromycin	GFP
pGIPZ shORP3#b	CTCCGGTTCTTGGAGAAACATA	Puromycin	GFP
pGIPZ shORP3#c	AGGAACTTAGAAGAAGCTGAA	Puromycin	GFP

LVRU6MH shScramble	GCTTCGCGCCGTAGTCTTA	Hygromycin	mCherry
LVRU6MH shORP3#a	GCGTGTCATGCTGAGTCTAGA	Hygromycin	mCherry
LVRU6MH shORP3#b	GCCGAAAGGCTACGAGCAATA	Hygromycin	mCherry
LVRU6MH shORP3#c	GGAGAAACATATGAATGTATT	Hygromycin	mCherry

Table III: guide RNA vectors and sequences

**Plasmid**	**Target Sequence**	**Selection marker**	**Other**
lentiCRISPRv2-gGFP	CAGGGTCAGCTTGCCGTAGG	Puromycin	None
lentiCRISPRv2-gORP3#1	AGTAGCTGCTCTTCCAAGCA	Puromycin	None
lentiCRISPRv2-gORP3#2	GCCCTTAAAAGGCTGGCATA	Puromycin	None

Table IV: Primers for qPCR

Target gene	Direction	Sequence 5’ - 3’	PCR product length (in bp)
ORP3	Forward	GTCATCCGCCCTAGCACAAAA	66
	Reverse	AGAGACTCGGCATGGATTCTG	
GAPDH	Forward	AAGGTCATCCCTGAGCTGAAC	142
	Reverse	ACGCCTGCTTCACCACCTTCT	

qPCR conditions

Stage	Step	Ramp rate	Temperature	Time
Hold stage	Step 1	1.6 °C/s	50 °C	2 mins
	Step 2	1.6 °C/s	95 °C	10 mins
PCR stage (40 cycles)	Step 1	1.6 °C/s	95 °C	15 sec
	Step 2	1.6 °C/s	60 °C	1 min
Melt curve	Step 1	1.6 °C/s	95 °C	15 sec
	Step 2	1.6 °C/s	60 °C	1 min
	Step 3	0.05 °C/s	95 °C	30 sec
	Step 4	0.05 °C/s	60 °C	15 sec
	Step 5	0.05 °C/s	4 °C	15 sec

Table V: Genotyping primers

**Program**	**Name**	**Sequence**
KRT14Cre	Genotype-KRT-Cre-F	GCCTGCAGGCCCACACCTCC
	Genotype-KRT-Cre-R	GTGTACGGTCAGTAAATTG
Orp3 (Osbpl3)	Osbpl3 F	CCAGCCACGAATCCTTCAC
	Osbpl3 R	CGGAGGACACTCAAAGAAGAGG
	CAS	TCGTGGTATCGTTATGCGCC
